# Eyes on the Line: A Case of Ocular Granulomatosis With Polyangiitis

**DOI:** 10.7759/cureus.37723

**Published:** 2023-04-17

**Authors:** Mani Maheshwari, Hemanthkumar Athiraman

**Affiliations:** 1 Hospital Medicine, Banner Health, Mesa, USA; 2 Hospital Medicine, Banner Health, Phoenix, USA

**Keywords:** c-anca/proteinase 3 (pr3)-positive granulomatosis with polyangiitis (gpa) formerly known as wegener's granulomatosis, rituximab therapy, granulomatosis with polyangiitis ( gpa), ocular manifestations, covid-19

## Abstract

Antineutrophilic cytoplasmic antibody (ANCA)-associated vasculitis is a small vessel vasculitis with a positive ANCA in the serum. One of three diseases that fall under this category is granulomatosis with polyangiitis (GPA), previously known as Wegener's granulomatosis. This case report presents a patient with an ocular manifestation of GPA, rendering a difficult diagnosis and multi-specialty approach to managing the disease.

## Introduction

Granulomatosis with polyangiitis (GPA) is a disorder that affects small arteries and veins through inflammation and necrotizing vasculitis [1]. Formerly known as Wegener's granulomatosis, GPA appears to be one of the most severe vasculitides [2]. It clinically affects the ear/nose/throat, lungs, and kidneys with the presence of antineutrophilic cytoplasmic antibody (ANCA) in 90% of systemic forms and the presence of proteinase 3 (PR3)-ANCA in 50% of localized forms [3]. In a survey of 701 patients, 30% of patients had ocular involvement [4]. In other surveys and studies, up to 50% of patients had ocular involvement, some having the ocular disease as the only complication of GPA [5-7]. This case report presents the ocular involvement of GPA in a 71-year-old female who eventually suffered vision loss.

## Case presentation

A 71-year-old female with a history of type 2 diabetes mellitus, end-stage renal disease due to ANCA-associated vasculitis and on hemodialysis, who was recently hospitalized for pneumonia secondary to *Acinetobacter baumannii,* *Aspergillus fumigatus*, and *Candida glabrata*, presented with right eye erythema, swelling, and pain for the past one month.

**Figure 1:**
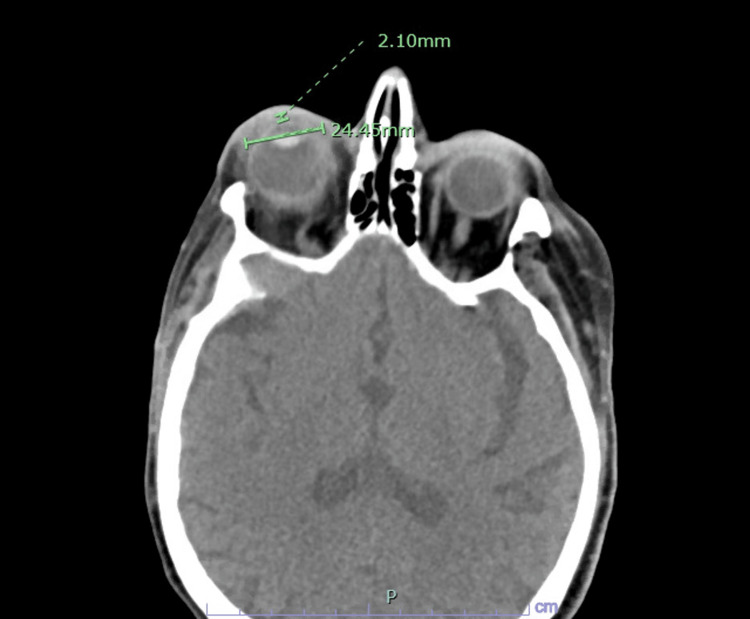
CT of the orbits shows right periorbital/palpebral soft tissue cellulitis with early abscess and diffuse wall thickening of the right optic globe sclera and cornea.

**Figure 2:**
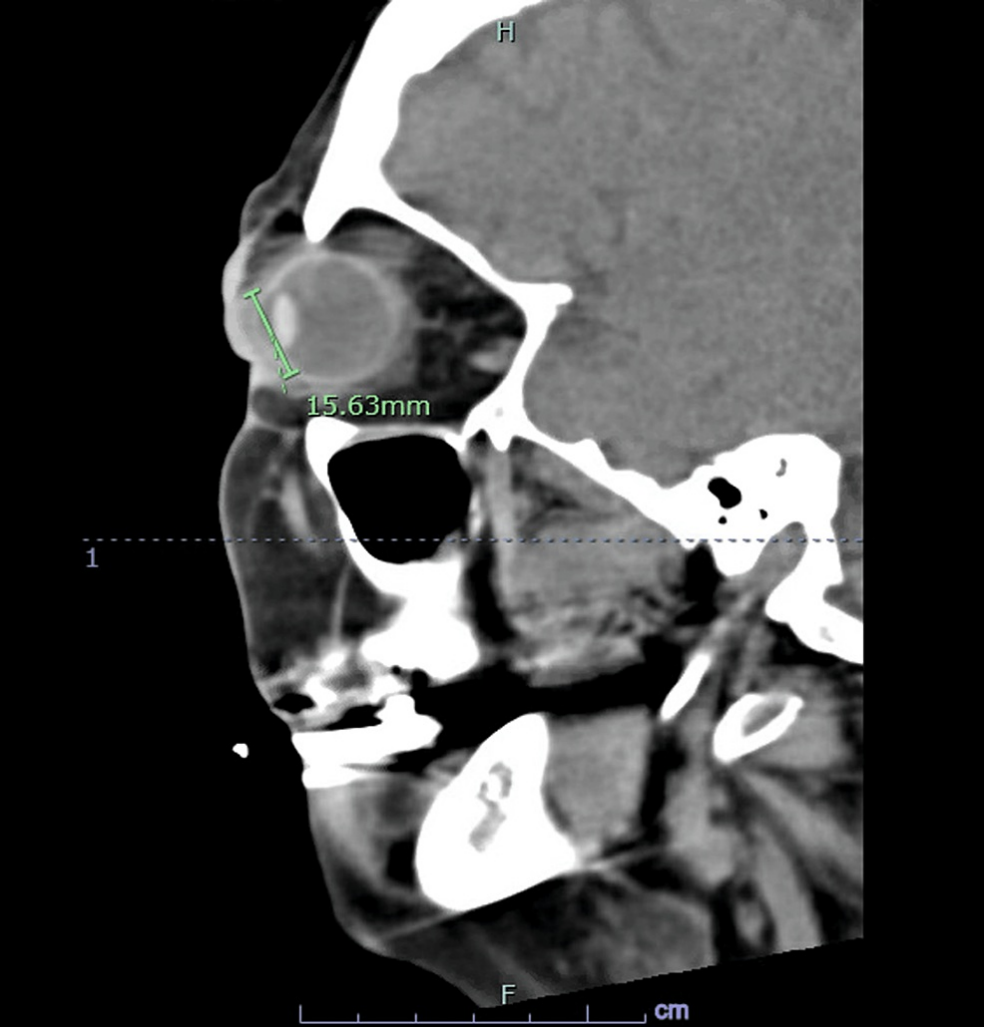
CT of the orbits shows stranding of retrobulbar intraconal fat and extraconal fat compatible with post-septal cellulitis and increased enhancement of the right optic nerve sheath complex and extraocular musculature, resulting in exophthalmos.

In the emergency department (ED), the patient’s vital signs were normal while the laboratory assessment revealed leukocytosis of 16.2 K/uL, elevated erythrocyte sedimentation rate (ESR) of 46 mm/hr, and renal function consistent with end-stage renal disease. A CT scan of the orbits showed right periorbital/palpebral soft tissue cellulitis with early abscess and diffuse wall thickening of the right optic globe sclera and cornea (Figure 1). Furthermore, there was stranding of retrobulbar intraconal fat and extraconal fat compatible with post-septal cellulitis and increased enhancement of the right optic nerve sheath complex and extraocular musculature, resulting in exophthalmos (Figure 2).

History-taking further revealed that the patient was recently prescribed prednisone and mycophenolate by her primary care physician. She was on rituximab in the past but developed multiple infections due to which it was discontinued. The patient was diagnosed with pulmonary tuberculosis (TB) two years before presentation and completed nine months of therapy with isoniazid. During the previous hospitalization for polymicrobial pneumonia, she had prolonged intubation with a failed extubation, after which she suffered cardiac arrest and had to be re-intubated followed by a tracheostomy. Post-discharge, she continued to take prednisone at home daily. Per charted history, the patient was admitted with ophthalmology and infectious disease consultations and started on IV daptomycin, meropenem, and voriconazole. Low-dose prednisone was continued.

Given her history, there was a concern for ANCA-associated vasculitis of the right eye, and she was transferred to a large university hospital where rheumatology is available. The rheumatologist’s workup revealed that the patient had highly positive titers for myeloperoxidase (MPO)/perinuclear anti-neutrophil cytoplasmic antibodies (P-ANCA), consistent with the diagnoses of GPA. She was started on pulse dose steroids (1 gm of Solu-Medrol) for three days. There was a consideration to restart rituximab; however, the patient was hesitant to get vaccinated (for pneumonia, influenza, and COVID-19). Furthermore, her chest X-ray showed left lower lobe consolidation. Given the possibility of recurrent pneumonia, she underwent a bronchoscopy from which bronchioalveolar lavage cultures revealed colonization with carbapenem-resistant* Klebsiella pneumoniae*. Given the patient's refusal to get vaccinated and the risk of TB reactivation, rituximab was not started at this point. She was discharged to a skilled nursing facility on a very long steroid taper of prednisone over 14 weeks with continued dosing of 6 mg of prednisone daily. However, she did not regain her vision.

The patient returned to the ED two weeks after for right eye pain. She was managed on prednisone and incidentally was also diagnosed with COVID-19 pneumonia. Her pneumonia worsened and led to respiratory failure. She ultimately required intubation and was placed on a mechanical ventilator. Despite multiple attempts and therapies to better COVID-19 pneumonia, the patient eventually passed from the same.

## Discussion

On average, once diagnosed with GPA, patients live only five more months without treatment [8]. Importantly, a multidisciplinary and multimodal approach ensures the benefit of affected organs and body systems [9]. The cornerstone treatment for GPA is the combination of corticosteroids and rituximab. This combo can relieve around 80% of GPA cases [3]. Unfortunately, however, the relapse is as high as 50% even in such cases [3]. Vision loss or blindness can be seen in as high as 37% of patients if there is a delay in diagnosis [10].

A presentation of ocular involvement may be misunderstood as being local but not systemic disease. However, this is not true [10]. Most commonly, scleritis or conjunctivitis will be observed and is followed by retro-orbital pseudotumor, keratitis, compressive neuropathy, and retinal vasculitis, along with other manifestations of the disease [11].

Similar to our patient, a trial involved a head-to-head comparison of rituximab and cyclophosphamide with glucocorticoids in patients affected with severe kidney disease (estimated glomerular filtration rate (eGFR)<30 [12]. In a head-to-head trial between rituximab and cyclophosphamide, both were part of a treatment regimen with glucocorticoids in a study of patients with severe kidney manifestation (eGFR<30), the same as in our patient [12]. Both groups had similar rates of remission, however, more patients achieved complete remission in the rituximab group indicating the ability to completely taper off prednisone therapy [13]. This is the best treatment thus far for GPA.

## Conclusions

It is crucial to remember the subtle manifestations of GPA and forms of the disease that one may think is "quiet," such as ocular disease. It is a difficult diagnosis and requires a multi-specialty approach to manage the disease. The earlier the treatment for GPA, the better the outcome.
